# OpenAnnotateApi: Python and R packages to efficiently annotate and analyze chromatin accessibility of genomic regions

**DOI:** 10.1093/bioadv/vbae055

**Published:** 2024-04-10

**Authors:** Zijing Gao, Rui Jiang, Shengquan Chen

**Affiliations:** Ministry of Education Key Laboratory of Bioinformatics, Bioinformatics Division at the Beijing National Research Center for Information Science and Technology, Center for Synthetic and Systems Biology, Department of Automation, Tsinghua University, Beijing 100084, China; Ministry of Education Key Laboratory of Bioinformatics, Bioinformatics Division at the Beijing National Research Center for Information Science and Technology, Center for Synthetic and Systems Biology, Department of Automation, Tsinghua University, Beijing 100084, China; School of Mathematical Sciences and LPMC, Nankai University, Tianjin 300071, China

## Abstract

**Summary:**

Chromatin accessibility serves as a critical measurement of physical contact between nuclear macromolecules and DNA sequence, providing valuable insights into the comprehensive landscape of regulatory mechanisms, thus we previously developed the OpenAnnotate web server. However, as an increasing number of epigenomic analysis software tools emerged, web-based annotation often faced limitations and inconveniences when integrated into these software pipelines. To address these issues, we here develop two software packages named OpenAnnotatePy and OpenAnnotateR. In addition to web-based functionalities, these packages encompass supplementary features, including the capability for simultaneous annotation across multiple cell types, advanced searching of systems, tissues and cell types, and converting the result to the data structure of mainstream tools. Moreover, we applied the packages to various scenarios, including cell type revealing, regulatory element prediction, and integration into mainstream single-cell ATAC-seq analysis pipelines including EpiScanpy, Signac, and ArchR. We anticipate that OpenAnnotateApi will significantly facilitate the deciphering of gene regulatory mechanisms, and offer crucial assistance in the field of epigenomic studies.

**Availability and implementation:**

OpenAnnotateApi for R is available at https://github.com/ZjGaothu/OpenAnnotateR and for Python is available at https://github.com/ZjGaothu/OpenAnnotatePy.

## 1 Introduction

The advancement of high-throughput sequencing technologies has yielded vast amounts of chromatin accessibility sequencing data, furnishing invaluable information for comprehending regulatory mechanisms (Klemm *et al.* 2019). Chromatin accessibility (openness) profiling holds broad applicability in studying cellular development, cell differentiation, regulatory mechanism, and human diseases. However, the process of annotating the accessibility of genomic regions can become cumbersome and time-consuming due to several manual and tedious steps, such as experiment identification and raw data downloading (Wang *et al.* 2021). Therefore, we have previously developed OpenAnnotate (Chen *et al.* 2021), a web server for efficiently annotating the openness of genomic regions. The web server, based on collected sequencing samples from ENCODE (Encode Project Consortium, 2012) and ATACdb (Wang *et al.* 2021), can annotate the openness of genomic regions for various biosample types, tissues, and biological systems.

Since its inception, OpenAnnotate has received extensive usage. Nevertheless, despite its efficacy in annotating openness across various biosample types, the web-based annotation approach faces several limitations. Firstly, OpenAnnotate presents each annotation task on an individual page, leading to a tedious process when annotating multiple genomic region files simultaneously. When users aim to acquire openness scores for dozens of cell types, it requires multiple submissions, which leads to efficiency constraints. Secondly, with the development of R and Python-based epigenomic data analysis software packages, the web server imposes inconvenience when applying annotations to other studies, as it necessitates manual file uploading and results downloading on the web page. The results from the web version of annotation still need to be manually converted into the data structures of various software pipelines. Thirdly, the current web version of OpenAnnotate lacks the capability to retrieve systems, tissues, and cell types hierarchically, resulting in inefficiency when selecting biosamples.

Meanwhile, recent advancements in single-cell sequencing technologies carry significant implications for comprehending cellular heterogeneity, developmental biology, and the mechanisms underlying diseases. Single-cell chromatin accessibility sequencing (scCAS) technologies enable the acquisition of chromatin accessibility information at the single-cell level, thereby providing the means to dissect cellular heterogeneity. RA3 (Chen *et al.* 2021) validated that bulk sequencing data has proven effective as reference data for supporting the characterization and imputation of scCAS data. Therefore, the annotations obtained from OpenAnnotateApi are of paramount utility in expediting the analysis of scCAS data. Presently, a plethora of tools with diverse functionalities are available for the analysis of scCAS data (Gao *et al.* 2023). Notable widely used tools include *EpiScanpy* (Danese *et al.* 2021), *ArchR* (Granja *et al.* 2021), *Signac* (Stuart *et al.* 2021), and *SnapATAC* (Fang *et al.* 2021). These widely used tools, predominantly implemented in Python or R, constitute the prevailing tools for the analysis of single-cell sequencing data. Therefore, for users of OpenAnnotate, there exists a demand for the development of an application programming interface (API) tool that can integrate with these platforms.

**Figure 1.**
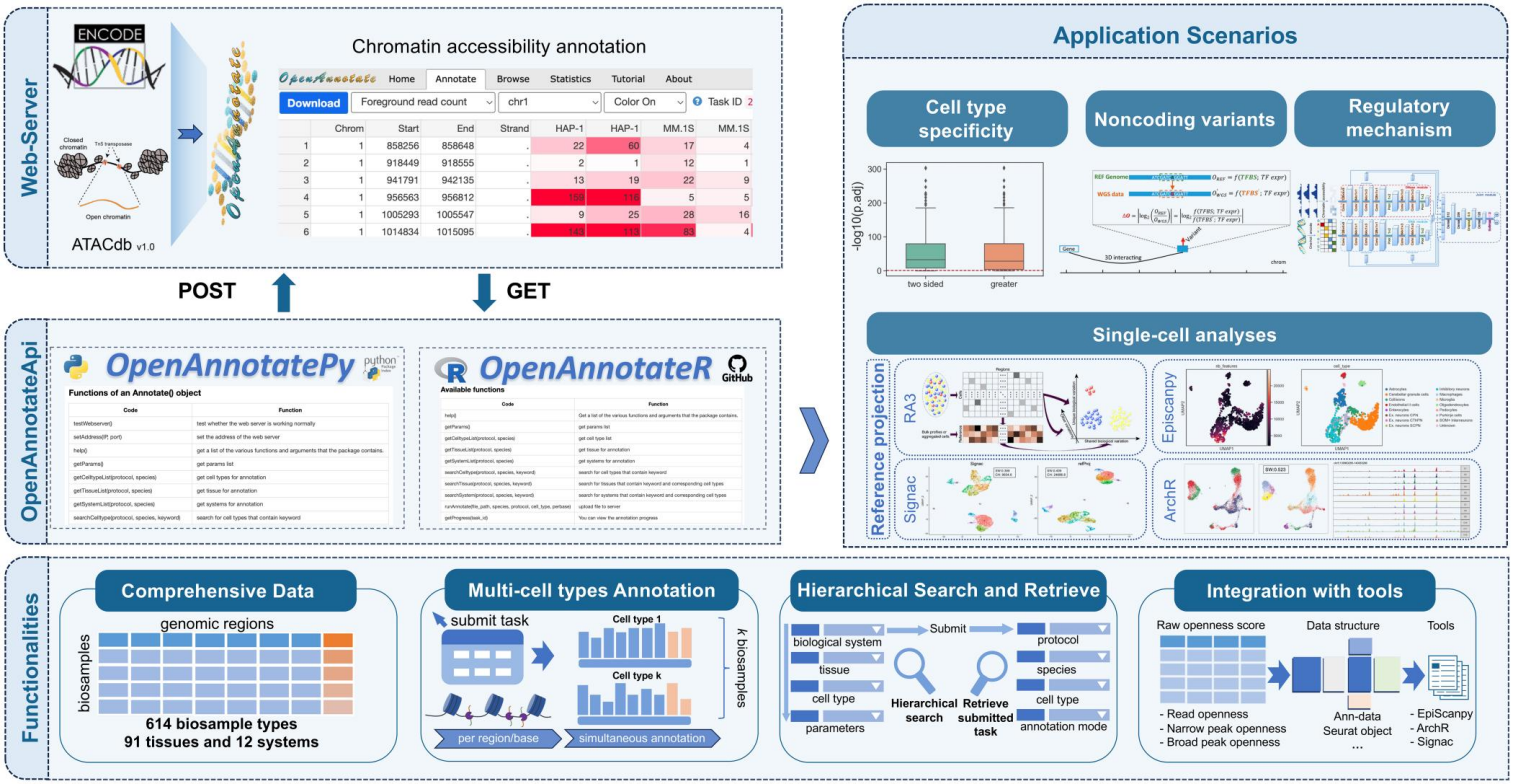
Overview of major features and application scenarios of OpenAnnotateApi. OpenAnnotateApi has extensive applications in cell type specificity, deciphering gene regulatory mechanisms, analyzing non-coding variations, and single-cell analysis. In single-cell analysis, OpenAnnotateApi demonstrates excellent compatibility with existing mainstream software tools, providing complementary external information for enhanced single-cell analysis.

To overcome the above limitations, we developed OpenAnnotateApi, comprising an R package (OpenAnnotateR) and a Python package (OpenAnnotatePy), as R and Python are two widely used platforms used in bioinformatics community (Fig. 1). OpenAnnotateApi efficiently bridges the communication between the client and the web server, containing a comprehensive set of functions that facilitating seamless annotation of openness of genomic regions and browsing the progress of annotation tasks (Supplementary Tables S1 and S2). Compared with web server, OpenAnnotateApi presents four notable enhancements (Supplementary Table S3). (i) OpenAnnotateApi offers functionalities allowing hierarchical searches within systems, tissues, and cell types for annotation. (ii) users can efficiently annotate the openness scores of the same set of genomic regions across multiple cell types simultaneously. (iii) the output results can be directly transformed into the data structures used by mainstream analysis pipelines, such as Ann-data in *EpiScanpy* (Danese *et al.* 2021), significantly enhancing the efficiency of the openness analysis. (iv) OpenAnnotateApi can be seamlessly applied to mainstream scCAS data analysis tools (Supplementary Texts S5, S6, and S8), including *Signac* (Stuart *et al.* 2021), *ArchR* (Granja *et al.* 2021), and *EpiScanpy* (Danese *et al.* 2021). In summary, OpenAnnotateApi offers researchers, particularly algorithm developers, a more convenient and efficient interface, substantially enhancing the functionality and usability of OpenAnnotate. We anticipate that OpenAnnotateApi will prove advantageous to both biologists and data scientists, enabling them to more effectively model the regulatory landscape of the genome.

## 2 Materials and methods

OpenAnnotateR is an R package for efficient annotation of the openness scores in genomic regions, developed and tested using the RStudio IDE with R 4.0.2. It functions as an API for the OpenAnnotate web server, utilizing the hypertext transfer protocol (HTTP) and relying on the *RCurl* and *httr* packages. The POST method is employed to request genomic region data in BED format and additional parameters from the local client to the server. Conversely, the GET method is utilized to retrieve data and annotate results from the server. Similarly, OpenAnnotatePy is a Python command-line tool intended for the same task, developed with Python 3.9 and the latest version released on the Python Package Index (PyPI). This package offers the same functions as OpenAnnotateR, facilitating easy integration into Python-based pipelines. Utilizing the *request* package, POST and GET methods are utilized to upload data to the server and download results from it (Supplementary Texts S1 and S2).

In the implementation of fundamental interface functionalities, both packages rely on the HTTP protocol. When executing any annotation task, the entire computational process can be delineated into the following three primary steps. Firstly, genomic regions, protocol specifications, species information, biosample types, and annotation modes are encapsulated into a multipart form, which is subsequently submitted to the server using the POST method. Secondly, once receiving the input data form, the server initiates the annotation program, storing the progress and results in specific files based on the task identifiers (task-id). Lastly, following task submission, users have the option to perform parameter queries. The downloading and conversion of annotation results become available upon the completion of the specific annotation task.

The core framework of both packages is based on the utilization of task-id to enable various functionalities. Upon submitting an annotation task to the server, each task is assigned a unique task-id. Subsequently, we have developed a series of functions utilizing the task-id for tasks such as real-time tracking of annotation progress, retrieving task parameters, and downloading annotation results. These functions efficiently locate and trace task-related information on the server. The backend server is constructed on a Linux-based Apache web server (v2.4.58). Computation tasks are executed using a parallel strategy, and the web server is deployed on a high-performance computing cluster (Supplementary Text S9). The robustness of the OpenAnnotateApi and its associated functions has undergone comprehensive testing on MacOS and Linux systems.

## 3 Results and applications

The main function of OpenAnnotateApi is to annotate chromatin accessibility across multiple cell types within arbitrary genomic regions at a whole-genome scale in a single submission. The pipeline of our packages encompasses the following five main functionalities.

### 3.1 Parameter query

Users have the capability to inquire about information regarding 2 species, 614 biosample types from 3 reference databases. OpenAnnotateApi offers retrieval at the system, tissue, and cell type hierarchy levels, so that users can efficiently query biosample types through keywords or fuzzy search, significantly improving retrieval efficiency.

### 3.2 Openness annotation

Users can accomplish annotation tasks by specifying the local path of a BED file or inputting data in Data Frame format. The task can be submitted with a one-click command *runAnnotate()*. Additionally, users have the option to choose between “per region” and “per base” annotation mode, thereby annotating chromatin accessibility at different resolutions (Supplementary Text S3).

### 3.3 Task information query

Following the submission of the annotation task, users can query the progress of the annotation and review the parameters set during task submission by retrieving the task-id.

### 3.4 Result downloading

Users can download the foreground read count and various openness scores generated under three different openness annotation approaches upon completion of the task (Supplementary Text S3).

### 3.5 Result converting

We have introduced data structure conversion functions to facilitate seamless integration into single-cell data analysis. For instance, within the Python package, a function has been included to convert results into the Ann-data format, while in the R version, a function has been added to transform results into the Seurat format.

Based on these functionalities, OpenAnnotateApi offers a broader range of application scenarios compared to the web server, including but not limited to cell type specificity analysis, single-cell analysis, regulatory element identification, and non-coding variant analysis (Supplementary Texts S4 and S5; Supplementary Figs S1–S4 and S6). Here, we describe two example application scenarios.

In the first scenario, if users want to investigate regulatory mechanisms based on chromatin accessibility of regulatory elements, particularly for predicting enhancers, they can integrate OpenAnnotateApi into their research workflow. For instance, DeepCAPE (Chen *et al.* 2021) is a convolutional neural network designed taking DNA sequences and DNase-based openness scores as inputs for enhancer prediction. Taking human epithelial cells of the esophagus as an instance, by utilizing OpenAnnotateApi, researchers can efficiently retrieve the chromatin accessibility (openness) features of genomic sequences. By combining these openness features with sequence characteristics, the DeepCAPE model achieves an average area under the receiver operating characteristic curve (auROC) of 0.979 in predicting human epithelial cell of esophagus enhancers under a 1:1 positive-to-negative sample ratio in 5-fold cross-validation. Nonetheless, when using only genomic sequence data for training, the DeepCAPE (seq-only) model only yields an average auROC of 0.725 (Supplementary Text S4 and Supplementary Fig. S6). Additionally, under sample ratios of 1:1, 1:2, and 1:3, the inclusion of the openness score all brings significant improvements, with an average increase of 21.6% and 36.3% in auROC and area under the precision–recall curve (auPRC). This indicates that the inclusion of openness scores can offer cell-type-specific insights to the model, thereby improving its ability to discriminate enhancer regulatory elements.

In the second scenario, if users aim to analyze scCAS data, the openness score proves to be a valuable reference for facilitating dimensionality reduction using a reference-guided projection approach (Supplementary Text S8). For example, in the context of exploring scCAS data of mouse cerebellum from the Mouse Cell Atlas (MCA) and utilizing *EpiScanpy* for data analysis (Cusanovich *et al.* 2018), researchers can leverage OpenAnnotatePy to obtain openness scores for 144 biosamples of each peak. Through applying principal component analysis (PCA) on the openness scores and subsequently using the learned projection vectors to project term frequency-inverse document frequency transformed scCAS matrix, the clustering performance can be significantly improved (Supplementary Fig. S1). We also validated the effectiveness of OpenAnnotateR in integration with other scCAS analysis pipelines based on R platform, such as ArchR and Signac. As an example, in Signac, utilizing the openness score for 871 biosamples for dimensionality reduction enhances the clustering performance on human peripheral blood mononuclear cells, with silhouette coefficient increased from 0.399 to 0.409. Similarly, in ArchR, upon incorporating the openness score, the silhouette coefficient of the clustering increased from 0.495 to 0.505 using the tutorial data (Supplementary Texts S5  and S6; Supplementary Figs S1, S3, and S4; Supplementary Table S4). In summary, bulk chromatin accessibility profiles can serve as external resources for the dimensionality reduction of scCAS data, allowing users to utilize bulk openness data as a reference for single-cell data analysis and explore various integration approaches, further constituting a crucial asset in single-cell epigenomics studies. The OpenAnnotateApi guarantee a user-friendly and efficient experience for researchers engaged in chromatin accessibility analysis.

Furthermore, we have validated the computational efficiency of OpenAnnotateApi, demonstrating its ability to efficiently process large-scale regions within a short timeframe. We collected over 500, 000 candidate silencer elements from SilencerDB (Zeng *et al.* 2021), with an average length of approximately 113 base pairs. The results indicate that annotating the accessibility of these 500, 000 regions can be completed in around 7 minutes. Annotating approximately 200, 000 regions per-base takes about 20 minutes. This efficiency represents a significant improvement compared to other annotation tools, such as CistromeDB (Zheng *et al.* 2019), which can annotate chromatin accessibility for only one genomic region at a time. To annotate genomic regions of such scale with CistromeDB, manual submissions would be required tens of thousands of times, each consuming several seconds of computing time. This computational efficiency falls significantly short compared to OpenAnnotateApi. We also verified the efficiency advantages of OpenAnnotateApi over web server in various scenarios (Supplementary Text S7 and Supplementary Fig. S7). This efficiency and computational capability form the foundational basis for its successful application in various other research domains (Supplementary Texts S7 and S9; Supplementary Figs S5 and S7).

## 4 Conclusion and discussion

In conclusion, OpenAnnotateApi comprises two toolkits, the R version and Python version, operating together as the command-line iteration of OpenAnnotate, which efficiently annotates chromatin accessibility signals across diverse biosample types. Besides, OpenAnnotateApi holds extensive applicability, particularly in single-cell data analysis. First, computer scientists can integrate openness scores from OpenAnnotateApi into models to predict and discover regulatory elements, and even construct regulatory networks. Second, openness scores provide valuable external information aiding single-cell data analysis. Third, openness scores offer a framework for interpreting and analyzing extensive genetic data from large-scale genome-wide association studies (Supplementary Text S4).

For future versions, OpenAnnotateApi has two potential directions for improvement. Firstly, with the advancement of multi-omics sequencing technologies, we are considering integrating DNA methylation data into OpenAnnotateApi in the future, aiming to annotate chromatin accessibility and methylation levels on a genome-wide scale. This will lead to a more exhaustive tool for annotating the epigenomic landscape. Secondly, with the advancements in single-cell and spatial sequencing technology, we contemplate incorporating single-cell scale chromatin accessibility data and organ-scale spatial epigenomic data into OpenAnnotateApi and OpenAnnotate, for a more comprehensive annotation of cellular chromatin accessibility across diverse tissues and cell types. We anticipate that OpenAnnotateApi will serve as a practical tool to facilitate the deciphering of gene regulatory patterns, exploration of cellular heterogeneity, and contribute to a deeper comprehension of the mechanisms underlying the development of human diseases.

## Supplementary Material

vbae055_Supplementary_Data

## Data Availability

The data underlying this article are available in the article and in its online supplementary material.

## References

[vbae055-B1] Chen S , Gan M, Lv H et al DeepCAPE: a deep convolutional neural network for the accurate prediction of enhancers. Genomics Proteomics Bioinformatics 2021;19:565–77. 33581335 10.1016/j.gpb.2019.04.006 PMC9040020

[vbae055-B2] Chen S , Liu Q, Cui X et al OpenAnnotate: a web server to annotate the chromatin accessibility of genomic regions. Nucleic Acids Res 2021;49:W483–90. 33999180 10.1093/nar/gkab337 PMC8262705

[vbae055-B3] Chen S , Yan G, Zhang W et al RA3 is a reference-guided approach for epigenetic characterization of single cells. Nat Commun 2021;12:2177. 33846355 10.1038/s41467-021-22495-4 PMC8041798

[vbae055-B4] Encode Project Consortium. An integrated encyclopedia of DNA elements in the human genome. Nature 2012;489:57. 22955616 10.1038/nature11247 PMC3439153

[vbae055-B5] Cusanovich DA , Hill AJ, Aghamirzaie D et al A single-cell atlas of in vivo mammalian chromatin accessibility. Cell 2018;174:1309–24.e18. 30078704 10.1016/j.cell.2018.06.052 PMC6158300

[vbae055-B6] Danese A , Richter ML, Chaichoompu K et al EpiScanpy: integrated single-cell epigenomic analysis. Nat Commun 2021;12:5228. 34471111 10.1038/s41467-021-25131-3 PMC8410937

[vbae055-B7] Fang R , Preissl S, Li Y et al Comprehensive analysis of single cell ATAC-seq data with SnapATAC. Nat Commun 2021;12:1337. 33637727 10.1038/s41467-021-21583-9 PMC7910485

[vbae055-B8] Gao Z , Chen X, Li Z et al scEpiTools: a database to comprehensively interrogate analytic tools for single-cell epigenomic data. J Genetics Genomics 2023; 10.1016/j.jgg.2023.09.011 37769837

[vbae055-B9] Granja JM , Corces MR, Pierce SE et al ArchR is a scalable software package for integrative single-cell chromatin accessibility analysis. Nat Genet 2021;53:403–11. 33633365 10.1038/s41588-021-00790-6 PMC8012210

[vbae055-B10] Klemm SL , Shipony Z, Greenleaf WJ. Chromatin accessibility and the regulatory epigenome. Nat Rev Genet 2019;20:207–20. 30675018 10.1038/s41576-018-0089-8

[vbae055-B11] Stuart T , Srivastava A, Madad S et al Single-cell chromatin state analysis with signac. Nat Methods 2021;18:1333–41. 34725479 10.1038/s41592-021-01282-5 PMC9255697

[vbae055-B12] Wang F , Bai X, Wang Y et al ATACdb: a comprehensive human chromatin accessibility database. Nucleic Acids Res 2021;49:D55–64. 33125076 10.1093/nar/gkaa943 PMC7779059

[vbae055-B13] Zeng W , Chen S, Cui X et al SilencerDB: a comprehensive database of silencers. Nucleic Acids Res 2021;49:D221–8. 33045745 10.1093/nar/gkaa839 PMC7778955

[vbae055-B14] Zheng R , Wan C, Mei S et al Cistrome data browser: expanded datasets and new tools for gene regulatory analysis. Nucleic Acids Res 2019;47:D729–35. 30462313 10.1093/nar/gky1094 PMC6324081

