# Supplementary material for: OpenAnnotateApi: Python and R packages to efficiently annotate and analyze chromatin accessibility of genomic regions
Source: Bioinform Adv. 2024 Apr 10;4(1):vbae055. doi: 10.1093/bioadv/vbae055 (PMC11031356; doi:10.1093/bioadv/vbae055)
Supplement: vbae055_Supplementary_Data [file vbae055_supplementary_data.pdf]

Supplementary Information

OpenAnnotateApi: Python and R packages to efficiently annotate and analyze chromatin accessibility of genomic regions

Zijing Gao[1], Rui Jiang[1] and Shengquan Chen[2,*]

[1]Ministry of Education Key Laboratory of Bioinformatics, Bioinformatics Division at the Beijing National Research Center for Information Science and Technology, Center for Synthetic and Systems Biology, Department of Automation, Tsinghua University, Beijing 100084, China and [2]School of Mathematical Sciences and LPMC, Nankai University, Tianjin 300071, China

*To whom correspondence should be addressed.

E-mail: chenshengquan@nankai.edu.cn

Contents

Supplementary Texts 3

Text S1. Design and implementation details of OpenAnnotatePy. 3

Text S2. Design and implementation details of OpenAnnotateR. 4

Text S3. The calculation of the openness score. 5

Text S4. OpenAnnotateApi facilitates studies of regulatory mechanism and noncoding variants. 6

Text S5. OpenAnnotateApi sheds light on single-cell data analyses. 10

Text S6. The evaluation metrics for clustering on single cell data. 12

Text S7. Computational efficiency of OpenAnnotateApi. 14

Text S8. Reference-guided projection method in single-cell analysis. 17

Text S9. The system design and computational capability of the backend web server. 19

Supplementary Figures 20

Fig. S1. OpenannotateApi is integrated into the analysis pipeline of Episcanpy. 20

Fig. S2. Cell-Type-Specificity of regulatory elements revealed by OpenAnnotateApi. 22

Fig. S3. OpenannotateApi is integrated into the analysis pipeline of Signac. 23

Fig. S4. OpenannotateApi is integrated into the analysis pipeline of ArchR. 24

Fig. S5. Computational efficiency of OpenAnnotateApi. 25

Fig. S6. The performance of DeepCAPE and DeepCAPE (seq-only) in predicting enhancers in epithelial cells of the esophagus. 26

Fig. S7. The distribution of the time taken for manually submitting annotation tasks to the web server 20 times (seconds). 27

Supplementary Tables 28

Table S1. Function names and functionalities of OpenAnnotatePy (As of Apr. 2024). 28

Table S2. Function names and functionalities of OpenAnnotateR (As of Apr. 2024). 29

Table S3. Comparison of functionalities in OpenAnnotateApi, OpenAnnotate and other toolkits. 30

Table S4. Summary of the scCAS datasets in single-cell data analyses with OpenAnnotateApi. 32

References 33

Supplementary Texts

Text S1. Design and implementation details of OpenAnnotatePy.

OpenAnnotatePy is a Python command-line tool intended for the annotation of the openness scores of genomic regions, developed with Python 3.9. The basic functionality of OpenAnnotatePy relies on communication with the backend server through Hypertext Transfer Protocol (HTTP). Both GET and POST methods are based on the Python package *requests* version 2.27.1. In terms of implementation logic, before submitting annotation tasks, all queries can be conducted without requiring a task ID. Utilizing the GET method allows querying various information on species, protocol, systems, tissues, and cell lines stored on the server. Additionally, it enables retrieval of example submission files from the server for local download. Furthermore, users can set task parameters based on queried information and genomic region files saved locally. Upon submission of an annotation task, each task and its detailed information are linked to a unique task ID. We package the input file data and parameters as a multipart form and upload it to the server using the POST method, then obtaining a unique task ID. Regarding result retrieval, all annotation results are processed in the server's backend, stored in specified paths based on the task ID. We access and download annotated results using the GET method, reading them into memory for processing and data structure conversion. All the functionalities described above have undergone comprehensive testing on MacOS and Linux systems to ensure robustness and compatibility.

Text S2. Design and implementation details of OpenAnnotateR.

OpenAnnotateR is an R package also intended for the annotation of the openness scores of genomic regions, developed with R 4.0.2 using the RStudio IDE. The basic functionality of OpenAnnotateR also relies on communication with the backend server through HTTP and both GET and POST methods are based on the R package *httr* version 1.4.4. The overall implementation logic remains consistent with OpenAnnotatePy, with the additional inclusion of the functionality to convert annotation results into Seurat objects. In this transformation, each column represents a cell type based on bulk sequencing data, serving as external reference data for single-cell analysis. Similarly, all the functionalities described above also have undergone comprehensive testing on MacOS and Linux systems to ensure robustness and compatibility.

Text S3. The calculation of the openness score.

Raw read openness score is calculated in the same manner as in OpenAnnotate (Chen, et al., 2021) within OpenAnnotateApi. Specifically, it can be computed as follows:

$$S = \frac{N/L}{M/W}$$

where L denotes the length of the given genomic region, M denotes the number of reads falling into the background region, and N denotes the number of reads falling into the region using BAM/SAM file of the sample from ENCODE (Consortium, 2012) or ATACdb (Wang, et al., 2021). The background-size W is set to 1 million base pairs.

Similarly, OpenAnnotateApi annotates narrow/broad peak openness for ENCODE (Consortium, 2012; Davis, et al., 2018; Gorkin, et al., 2020) samples and ATACdb (Wang, et al., 2021) peak openness for ATACdb samples by calculating the ratio between the average number of peaks overlapping with the specific region of size R and the average number of peaks overlapping with a background region of size B surrounding the given genomic region. Alongside the two openness scores based on reads and peaks, OpenAnnotateApi also provides the foreground read count for alternative utilization.

Based on the aforementioned computational approach, we provide two annotation modes: 'Per region' and 'Per base', for annotations at different resolutions. The 'Per region' annotation mode calculates the openness score for each region in the input BED file, whereas the 'Per base' mode calculates the openness score for each base pair covered by all regions in the input BED file. This means setting L as 1, representing each base pair within the region. Therefore, the 'Per base' mode yields a much larger output compared to the number of regions. In the tutorial provided on the GitHub page of OpenAnnotateR, we utilized a BED file consisting of 640 genomic regions as input and selected the 'Per base' annotation mode to annotate all cell types. Thus, the output file such as read-open matrix contains 1,274,837 rows, representing the total length of the genomic regions covered by these 640 genomic regions. In the tutorial provided on the GitHub page of OpenAnnotatePy, we utilized the same BED file and selected the annotation mode as 'Per region'. Thus, the output file contains 640 rows, representing the openness score of 640 genomic regions. More details can be found in the web page of OpenAnnotate (http://health.tsinghua.edu.cn/openannotate/).

Text S4. OpenAnnotateApi facilitates studies of regulatory mechanism and noncoding variants.

OpenAnnotateApi facilitates a more convenient integration into the analysis pipeline concerning regulatory mechanisms. Specifically, openness scores provide valuable information regarding the accessibility of regulatory element sequences, contributing to cell-specific analysis of regulatory elements, identification of regulatory elements, recognition of regulatory relationships, and co-open analysis, same as OpenAnnotate (Chen, et al., 2021). The following applications have partly been covered in OpenAnnotate, and we conducted new experiments focusing on the cell-specific analysis of regulatory elements and identification of regulatory elements.

Cell type-specificity of regulatory elements

The openness annotation by OpenAnnotateApi can offer crucial cell type-specific patterns. It's been instrumental in showcasing the cell type-specificity of validated silencers (Zeng, et al., 2021) and in modeling how the openness of a regulatory element relies on its DNA sequence and TF expression (Li, et al., 2020). This modeling helps score the cell type-specific impacts of noncoding variants in personal genomes.

More methodological details can be found on the OpenAnnotate web page (http://159.226.47.242:65533/openness/anno/info/demos/CellTypeSpecificity/CellTypeSpecificity-python.html).

Compared to OpenAnnotate, OpenAnnotateApi integrates more directly and efficiently into Python and R scripts for analyses. As shown in Fig. S2, we utilized 6,924 silencers on K562 cell line and 1,679 silencers on HepG2 cell line from SilencerDB (Zeng, et al., 2021), all validated through high-throughput or low-throughput sequencing techniques.

To validate the effectiveness of openness in revealing the cell type specificity of regulatory elements, the experiment is primarily divided into three steps. Firstly, we downloaded the region of 6,924 silencers from SilencerDB (Zeng, et al., 2021) on the K562 cell line, all of which have been validated through high-throughput or low-throughput sequencing experiments. Secondly, we utilized the OpenAnnotateApi to annotate the openness of these regions in different cell lines or tissues, thereby observing the differences in chromatin accessibility distribution on the same genomic regions across different cell lines. Thirdly, using

read-open score as an example, we conducted hypothesis testing on the distribution of openness scores of these regions in the K562 cell line and other cell lines. We established hypotheses for the median of the openness score distribution and employed one-sided and two-sided Wilcoxon tests to verify two questions:

1) Whether the openness scores of regulatory elements in the K562 cell line are different from those in other cell lines.

$$H_0: m_K = m_O, H_1: m_K \neq m_O$$

2) Whether the openness scores of regulatory elements in the K562 cell line are greater than those in the majority of other cell lines.

$$H_0: m_K = m_O, H_1: m_K > m_O$$

Where m_K denotes the median openness score of silencers in the K562 cell line, and m_O denotes the median openness score of the same silencers in other cell lines.

Then we conducted the above hypothesis test for each comparison between the K562 cell line and every other cell line or tissue. Therefore, we obtained a total of 198 sets of hypothesis test results and corresponding p-values. As shown in Fig. S2, the results demonstrate that in the K562 cell line, the openness score distribution of silencer elements significantly differs from that of 188 other cell lines and is significantly higher than that of 163 of them. Similarly, in the HepG2 cell line, the openness score distribution of silencer elements significantly differs from that of 140 other cell lines and is significantly higher than that of 111 of them (p-value < 0.05).

More details on methodology can be found on the SilencerDB web page (http://health. tsinghua.edu.cn/silencerdb/analysis.php).

Identification of regulatory elements

Chromatin openness scores serve as crucial features of sequences, aiding in the discovery of potential regulatory elements such as promoters, enhancers, and silencers, which predominantly reside within chromatin accessible regions, providing additional information for element identification. DeepCAPE (Chen, et al., 2021) employs a convolutional neural network that takes DNA sequences and DNase-based openness scores as inputs to predict potential enhancer elements. This approach demonstrates significant performance improvement compared to models solely based on sequence information.

In specific, cross-validation experiments were conducted for predicting enhancers across multiple cell lines in DeepCAPE (Chen, et al., 2021). Additionally, a variation of our model, named "DeepCAPE (seq-only)," was proposed, which excluded the auto-encoder and DNase modules and predicted enhancers solely based on DNA sequence information. DeepCAPE (seq-only) demonstrated superiority over three baseline methods in most cases at sample ratios of 1:10 and 1:20. However, it still showed some performance gap compared to DeepCAPE incorporating the openness score. This demonstrates that DeepCAPE (seq-only) possesses the ability to identify enhancers but with limited performance. On the homepage of OpenAnnotate, a specific example is showcased wherein the identification of 166 human epithelial cell of esophagus enhancers at a positive-to-negative sample ratio of 1:5 was performed. Results from a single random sampling experiment indicated that DeepCAPE (seq-only) achieved an auROC of 0.5 and an auPRC of 0.167, whereas DeepCAPE achieved an auROC of 0.978 and an auPRC of 0.952.

The experimental results provided as an example exhibit a certain degree of randomness. Therefore, we conducted additional experiments with more balanced positive-to-negative sample ratios to validate the predictive capability of the DeepCAPE model and the DeepCAPE model using only DNA sequences as input (DeepCAPE seq-only). Specifically, we utilized 166 human epithelial cell of esophagus enhancers as positive samples, and randomly sampled DNA fragments of variable length from the background genome as negative samples, with the constraint that the length and GC content of negative samples should be identically distributed as those of known enhancers. We conducted 5-fold cross-validation with positive-to-negative sample ratios of 1:1, 1:2, and 1:3. The results showed that DeepCAPE (seq-only) achieved average auROC of 0.725, 0.766, and 0.788, and auPRC of 0.713, 0.584, and 0.524 for the three sample ratios (Fig. S6). Overall, the results demonstrates that the predictive performance of DeepCAPE (seq-only) significantly surpasses random guessing. Furthermore, through this case study, we emphasize the importance of the openness score for deep learning models in identifying enhancers. As shown in Fig. S6, the auROC and auPRC of DeepCAPE were both above 0.95 for all three sample ratios, and outperformed DeepCAPE (seq-only) by an average of 21.6% and 36.3% in terms of auROC and auPRC, demonstrating a more pronounced advantage under imbalanced positive-to-negative sample ratios. When predicting enhancers, if we aim to screen enhancer elements from the whole genome, we are faced with a situation of extremely low positive-to-negative sample ratio. This once again confirms the role of openness score.

More details on methodology can be found on the OpenAnnotate web page (http://159.226.47.242:65533/openness/anno/info/demos/RegulatoryMechanism/RegulatoryMechanism.html).

Identification of chromatin conformation

In addition to the prediction of regulatory elements on the genome, chromatin openness scores also contribute to studying the 3D interactions between the regulatory elements. DeepTACT (Li, et al., 2019) utilizes DNA sequence and openness scores, and neural networks to predict potential regulatory relationships between elements, such as the regulatory association between enhancers and promoters. This underscores the significance of openness annotations provided by OpenAnnotateApi in comprehending regulatory mechanisms.

Co-open regions analysis

Cell-type-specific openness scores have also been successfully applied in the investigation of gene co-opening networks, revealing the elucidation of gene functional relationships (Li, et al., 2017). Additionally, analyzing the co-accessibility of regulatory regions and neighboring genes aids in uncovering the regulatory functions of transcription factors (Song, et al., 2019).

Noncoding variants analysis

The openness annotation provided by OpenAnnotate has been applied to model the dependency of a regulatory element's openness score on both its underlying DNA sequence and the expression of transcription factors. This modeling approach enables the scoring of cell type-specific effects of noncoding variants in personal genomes (Li, et al., 2020).

Text S5. OpenAnnotateApi sheds light on single-cell data analyses.

In addition to the analysis of gene regulatory mechanisms, incorporating external reference information through openness scores in single-cell analysis enhances more precise dimensionality reduction, clustering, and identification of cell types within single-cell data. The annotation method of openness scores provided by OpenannotateApi has been successfully applied in various single-cell analysis research works. For example, RA3 (Chen, et al., 2021) characterizes single-cell data with the help of bulk chromatin accessibility sequencing data. RefTM (Zhang, et al., 2023) also utilizes bulk chromatin accessibility data and models single-cell chromatin accessibility data based on topic modeling.

Moreover, we specifically explored the functionality of OpenAnnotateApi as a command-line tool in single-cell analysis workflows. Utilizing the refProj method described in OpenAnnotate, we successfully integrated bulk chromatin accessibility data into the analysis pipelines of Episcanpy (Danese, et al., 2021), Signac (Stuart, et al., 2021), and ArchR (Granja, et al., 2021). This integration can provide additional information for the dimensionality reduction of single-cell chromatin accessibility (scCAS) data, thereby offering more comprehensive information for clustering analysis and possessing the potential to enhance clustering performance. Next, we will describe three examples to illustrate how to integrate bulk openness scores into these pipelines.

Episcanpy

Episcanpy is a Python toolkit for analyzing single-cell epigenomic data, encompassing processes such as data preprocessing, quality control, dimensionality reduction, clustering, visualization, and downstream analysis. In the dimensionality reduction step, we integrated OpenAnnotateApi using the refProj method with the peak openness scores before embedding it into Episcanpy (Text S8). Leveraging PCA based on 436,206 peaks on bulk open chromatin data from 144 biosamples using the mouse mm9 reference genome, we projected this information onto single-cell data for dimensionality reduction of single-cell chromatin accessibility data. Utilizing 2,278 cells in the cerebellum region from the MCA dataset (as shown in Fig. S1), employing both Louvain and Leiden clustering methods, our integration of OpenannotateApi demonstrated significantly enhanced efficacy in dimensionality reduction and clustering. For example, when using Leiden clustering, the adjusted mutual information (AMI) score increased from 0.624 to 0.677, homogeneity improved from 0.542 to 0.588, and the v-measure rose from 0.667 to 0.720. This illustrates that OpenAnnotateApi not only

seamlessly integrates into single-cell data analysis but also substantially contributes to the accuracy of single-cell data analysis.

Signac

Signac is an R package designed for analyzing single-cell epigenomic data. We also validated the effectiveness of integrating OpenAnnotateR into the Signac analysis workflow. Using 8,728 human peripheral blood mononuclear cells (PBMCs) provided by 10x Genomics from the tutorial data of Signac, we annotated 87,561 peaks on these cells and obtained bulk openness scores for 871 biosamples using the human hg19 reference genome. Using refProj method, we obtained the low-dimensional representation of cells. As shown in Fig S3, leveraging this low-dimensional representation improved cell clustering, resulting in higher average silhouette width scores (SW) and Calinski-Harabasz index (CH).

ArchR

Similarly, we validated the effectiveness of integrating OpenAnnotateR into ArchR's analysis pipeline. ArchR is an R package used for analyzing single-cell chromatin accessibility data based on fragment files. Specifically, we utilized 4,932 bone marrow mononuclear cells (BMMC), 2,454 peripheral blood mononuclear cells (PBMC), and 3,275 CD34+ hematopoietic stem and progenitor cells from bone marrow (CD34 BMMC) in the tutorial dataset generated by previous study (Granja, et al., 2019). The tutorial provided by ArchR automatically generated a matrix containing 6,072,620 genomic bins and selected 25,000 genomic bins for dimensionality reduction (https://www.archrproject.com/articles/Articles/tutorial.html).

Using the openness score of 25,000 bins across 871 biosamples selected genomic bins as a reference (using the human hg19 reference genome), our method reduced the clustering SW from 0.495 to 0.505, as shown in Fig. S4. This further underscores the capability of our analytical tool for assisting in the analysis of single-cell chromatin accessibility data derived from fragment inputs.

Text S6. The evaluation metrics for clustering on single cell data.

We used a total of 5 metrics to evaluate the labeled clustering results and employed two metrics for the unlabeled clustering evaluation. The ARI measures the similarity between the true cell type and the clustering result, we calculated it as

$$ARI = \frac{RI(T,P) - E\big(RI(T,P)\big)}{max\big(RI(T,P)\big) - E\big(RI(T,P)\big)}$$

where T denotes true labels of cells, P denotes predicted labels via clustering. RI is a measure of the similarity between two lists of labels. AMI is calculated as

$$AMI = \frac{MI(T,P) - E\big(MI(T,P)\big)}{avg\big(MI(T,P)\big) - E\big(MI(T,P)\big)}$$

where MI(·,·) denote the mutual entropy, avg(·) denote the average function, and E(·) denote the expectation function. Homogeneity is calculated as

$$Homo = 1 - \frac{H(T|P)}{H(T)}$$

where H(·) denote the entropy function and H(T|P) is the uncertainty of true labels based on the knowledge of predicted assignments. Fowlkes mallows is defined as

$$FMI = \frac{TP}{\sqrt{(TP + FP) \times (TP + FN)}}$$

where the *TP* denotes number of true positive, *FP* denotes number of false positive, and *FN* denotes number of false negative. Silhouette score (average silhouette width) is mean Silhouette Coefficient of all samples. The silhouette coefficient is calculated as

$$SC = \frac{b - a}{\max\{a, b\}}$$

where *a* denotes the mean intra-cluster distance and *b* denotes the mean nearest-cluster distance for a specific sample. V measure score is calculated as

$$v\ measure = \frac{(1 + \beta) * homogeneity * completeness}{(\beta * homogeneity + completeness)}$$

$$completeness = 1 - \frac{H(P|T)}{H(P)}$$

where β is the ratio of weight attributed to homogeneity versus completeness, set to 1 in our experiments. Calinski–Harabasz index is calculated as

$$CH = \frac{BCSS/(k-1)}{WCSS/(n-k)}, BCSS = \sum_{i=1}^{k} n_i \|c_i - c\|^2, \ \ WCSS = \sum_{i=1}^{k} \sum_{x \in C_i} \|x - c_i\|^2$$

where k denotes the number of clusters, n denotes the total data samples, c_i is the centroid of the ith cluster C_i, and n_i denotes the number of samples in the ith cluster. BCSS represents the between-cluster separation and WCSS represents the within-cluster dispersion.

Text S7. Computational efficiency of OpenAnnotateApi.

OpenAnnotateApi allows simultaneous annotation across multiple cell types and genomic regions, significantly enhancing annotation efficiency. In comparison, the annotation capability of CistromeDB (Zheng, et al., 2019) is restricted, as it annotates only one region for chromatin accessibility at a time, and the annotation of the peak set has not been organized into the form of openness for each individual peak. This limitation severely impacts annotation speed for large-scale datasets, requiring manual submission of multiple tasks. Using a file containing 339 candidate enhancer regions in acinar cells from FANTOM5 (Andersson, et al., 2014), OpenAnnotateApi completes per-base annotation in 5.18 seconds and per-base annotation in 10.69 seconds based on the OpenAnnotate web-server. In contrast, CistromeDB requires 339 submissions to annotate these regions. We sampled 20 regions and recorded the annotation time for each, averaging 7.31 seconds per annotation task. This implies approximately 2,479 seconds for all annotations on the region-level, surpassing OpenAnnotateApi's completion time by a hundredfold. If users intend to acquire the accessibility for each base pair (bp), CistromeDB demands hundreds of manual submissions for regions spanning several hundred base pairs, making it excessively time-consuming and labor-intensive. Even upon submitting an interval, further refinement of the output results are still necessary to obtain the accessibility score for each base. These results demonstrate the efficiency of OpenAnnotateApi in annotations and its heightened adaptability for downstream analysis.

Furthermore, we recorded the annotation times of OpenAnnotateApi on datasets of different scales. We recorded the annotation time for both region-level and per base-pair-level. Specifically, we downloaded approximately 500,000 potential silencer regulatory element regions from SilencerDB, with an average length of 113.5 bp. For region-based annotation, we randomly sampled 1,000, 5,000, 10,000, 50,000, 100,000, 200,000, 300,000, 400,000, and 500,000 regions for annotation across 871 biosamples. Notably, we observed that annotation for as many as 500,000 regions could be completed in approximately 7 minutes. Concerning per base annotation, we randomly sampled 1,000, 5,000, 8,000, 10,000, 20,000, 30,000, 50,000, 100,000, and 200,000 regions. Even with up to 200,000 regions and annotating an average of approximately 100 base pairs, the annotation could be completed in about 20 minutes (Fig. S5). This demonstrates the efficiency of OpenAnnotateApi in annotating large-scale data. Particularly in single-cell analyses where handling hundreds of thousands of peaks or tens of thousands of genomic regions is common, the validated computational efficiency further

underscores the utility of OpenAnnotateApi, not only enhancing performance but also achieving efficiency in computational tasks integrated within single-cell analysis.

Compared with using the web server, the API packages will demonstrate computational efficiency advantages in the following three scenarios:

1) When submitting annotation tasks for different genomic regions multiple times. Taking the annotation of openness score for regulatory element regions as an example, suppose the user has collected the regions of Silencers or enhancers in *K* different cell lines or tissues, and the user wants to observe the overall distribution of openness for regulatory elements in each cell line or tissue. If using the website, it would require manually storing these regions, entering the website to manually upload files for annotation, and waiting for results for the next step. In contrast, using the API allows direct submission and result downloading through code, followed by analysis of the results with code. A single submission process may save several seconds or minutes of time. For example, when conducting enhancer prediction experiments using DeepCAPE (Chen, et al., 2021) under a 5-fold cross-validation setting, involving the partitioning of training and testing samples as well as positive and negative samples, annotation of 20 different BED files is required. If utilizing the web server, manual submission of 20 tasks is necessary. We recorded an average submission time of 13.872 seconds per task, resulting in a total of 4.61 minutes for 20 repeated submissions (Fig. S7). Considering the waiting time, the additional time consumed would be even longer. If repeating experiments in more experimental settings, the time required would increase significantly, and the error rate for manual operations such as file submission would rise. In contrast, OpenAnnotateApi allows users to submit all tasks at once through code, eliminating the need for repetitive manual file and parameter selection operations.

2) In Python or R-based analysis workflows. Taking integration with single-cell data analysis pipelines as an example, suppose a user needs to annotate the openness score of peak/region in an scATAC-seq dataset in their research. For instance, users may find it difficult to directly access the web server through a browser on a command-line-based computational server. Instead, they need to download the peak/region files to their local computer, annotate them using the web server, download the result files to local computer, and then upload the result files back to the computational cluster server for analysis. Using OpenAnnotateApi not only saves several minutes of time but also enhances the coherence of the analysis, especially when dealing with multiple files.

3) When annotating the openness scores for multiple different cell types simultaneously for the same region, the efficiency advantage of the API package becomes even more pronounced. For example, if we aim to investigate the openness of candidate regulatory element regions in cell types such as HepG2, K562, GM12878, and A549. Using the web server would require four separate manual task submissions, followed by downloading and integrating the results. In contrast, utilizing the API package would only necessitate a single task submission. By selecting the four cell lines through parameters, we can directly obtain the corresponding results, significantly improving time efficiency.

Text S8. Reference-guided projection method in single-cell analysis.

The Openness scores for various cell types, obtained through OpenAnnotate or OpenAnnotateApi annotation, can serve as reference bulk data to aid in the analysis of scCAS data, such as dimensionality reduction and clustering. We employed a method called reference-guided projection (refProj) to assist in single-cell data analysis, which was previously proposed in our published work, OpenAnnotate (Chen, et al., 2021) and has been applied in RA3 (Chen, et al., 2021) and RefTM (Zhang, et al., 2023). More methodological details can also refer to these works.

In the application scenarios depicted in Fig. S1, S3, and S4, we utilized principal component analysis (PCA) and employed bulk openness data as a reference to reduce the dimensionality of scCAS data. Specifically, we first apply term frequency-inverse document frequency (TF-IDF) transformation to the scCAS count matrix or binarized matrix $A \in \mathbb{R}^{p\times n}$ as

$$TFIDF(A_{ij}) = \log\left(1 + \left(TF(A_{ij}) \times IDF(A_{ij})\right) \times 10^4\right), TF(A_{ij}) = \frac{A_{ij}}{\sum_i A_{ij}}, IDF(A_{ij}) = \frac{N}{\sum_j A_{ij}}$$

which represents how important the peak *i* is for cell j, where *n* represents total number of cells in the dataset and the IDF represents the inverse frequency of each peak across all cells.

Next, we save these *p* features/regions as a bed-format file and annotate the openness scores of *m* biosamples from the bulk sequencing data using OpenAnnotateApi with the corresponding reference genome with 'Per region' mode, resulting in a bulk-level openness score matrix $O^{p\times m}$, which represents the same p features/regions for the total of m biosamples. Then, we utilize PCA to perform dimensionality reduction on $O^{p\times m}$ to obtain latent variables for each biosample.

$$Z_b = W_b \times O$$

Where $Z_b \in \mathbb{R}^{k_1\times m}$ represents the latent variables obtained by applying PCA on the reference data, $W_b \in \mathbb{R}^{k_1\times p}$ is the projection matrix of PCA learned on the reference data.

Then, we apply the projection matrix W to the single-cell count matrix A as follows.

$$Z_1 = W_b \times TF \cdot IDF(A)$$

$Z_1 \in \mathbb{R}^{k_1\times n}$ utilizes prior information from the reference data and captures the shared biological variation among scCAS data and the reference data. We further utilize the dimensionality reduction method of the scCAS analysis tool to reduce the dimensionality of the scCAS count matrix, obtaining another reduced matrix $Z_2 \in \mathbb{R}^{k_2\times n}$, which represents the biological variation from only scCAS data. Finally, we concatenate the two matrices to represent the low-dimensional representation of cell j as

$$z_j = [z_{1j,} z_{2j}]$$

where z_{1j} represents a k_1-dimensional vector, z_{2j}represents a k_2-dimensional vector, and z_j represents the low-dimensional representation of jth cell after incorporating bulk openness scores. We further utilize z_j for clustering, visualization, and other downstream analyses. When integrating the reference method into Episcanpy (Danese, et al., 2021), Signac (Stuart, et al., 2021), and ArchR (Granja, et al., 2021), we replaced the original dimensionality reduction matrix in the single-cell analysis pipeline with the low-dimensional representation obtained using the reference-guided method described above.

Text S9. The system design and computational capability of the backend web server.

OpenAnnotateApi and OpenAnnotate (Chen, et al., 2021) both rely on the same computing cluster. Our tool provides computational services without requiring any local computing resources from the users. The current version of OpenAnnotate (Chen, et al., 2021) is deployed on a high-performance computing cluster, consisting of a calculation node and a server node. The calculation node is equipped with two Intel Scalable processors (with a total of 56 hyper-threads), 768 GB of RAM, and 480 TB of storage space.

Taking our provided EXAMPLE.bed file as an example, which contains 640 genomic regions, annotating these regions on the mode of region-based annotation requires a peak memory usage of approximately 86MB and takes about 10 seconds. Roughly estimating based on memory, the computing cluster has the capacity to simultaneously annotate approximately 89 files of this kind. From the CPU perspective, the peak CPU occupancy of such a process is 4, thus it can be estimated that it can efficiently annotate approximately 14 files of this kind simultaneously. If the restriction on CPU computing rate is relaxed, this estimation would be higher. Therefore, the current web server can meet the computational demands of high concurrent task numbers.

Additionally, we have compressed the annotation result files, greatly reducing the size of files that users need to download on the web-server. For example, for the annotation results of the example task ID provided on the web page, the size of the original raw read openness file is 166.4 MB, while the size of the compressed file that users need to download is only 61.8 MB. If annotating per base for over 240 million sites on the entire chromosome 1, the original file size would reach 456 GB, whereas the compressed file size that users need to download is only 17.6 GB. Therefore, the compression of results on the web-server can significantly reduce the time users spend on downloading.

Supplementary Figures

Fig. S1. OpenannotateApi is integrated into the analysis pipeline of Episcanpy.

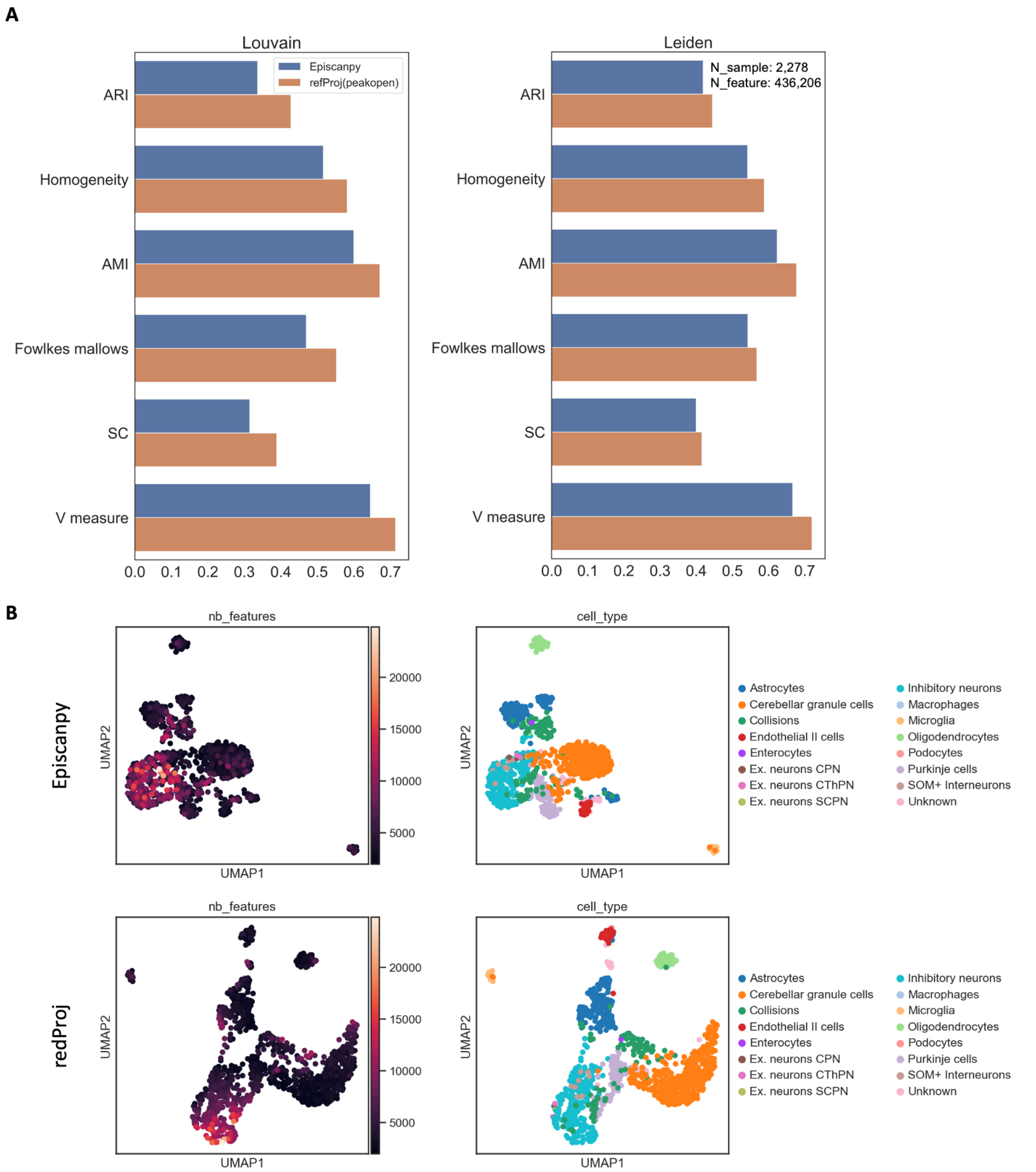

Fig. S1. OpenannotateApi is integrated into the analysis pipeline of Episcanpy. (A) Clustering performance of the original dimensionality reduction methods in Episcanpy and the refproj dimensionality reduction method integrated with OpenannotateApi on 2,278 mouse cerebellum cells with 436,206 peaks

(Left: Louvain, Right: Leiden). (B) Visualization of EpiScanpy and refProj dimensionality reduction and clustering (based on Louvain clustering).

Fig. S2. Cell-Type-Specificity of regulatory elements revealed by OpenAnnotateApi.

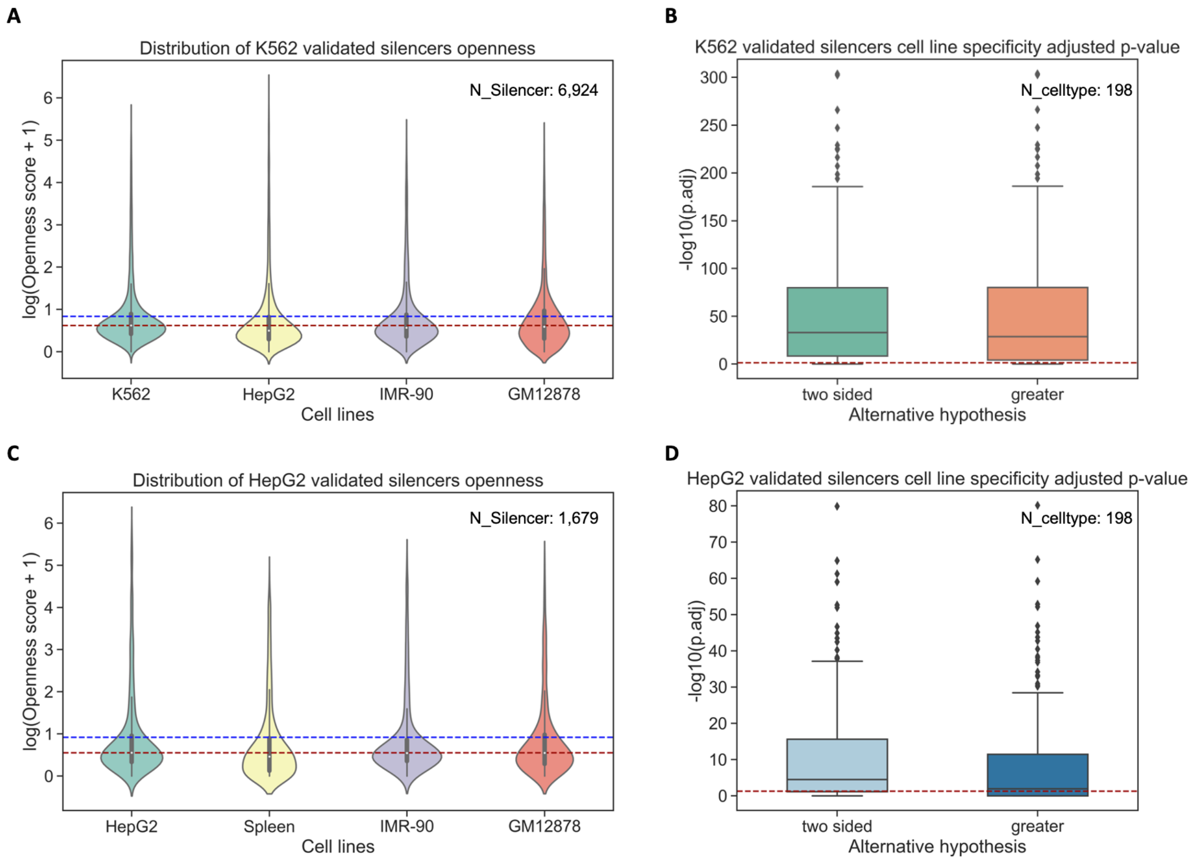

Fig. S2. Cell-Type-Specificity of regulatory elements revealed by OpenAnnotateApi. (A) The distribution of openness scores across different cell types on 6,924 experimentally validated silencers in K562 cell line (the blue dashed line denotes the mean of the silencer openness score on K562, and the red line denotes the median). (B) The distribution of cell-specific p-values for two-sided and one-sided Wilcoxon hypothesis tests of openness scores of silencers between K562 cell line and other 198 cell types (significantly lower than 0.05). (C) The distribution of openness scores across different cell types on 1,679 experimentally validated silencers in HepG2 cell line (the blue dashed line denotes the mean of the silencer openness score on HepG2, and the red line denotes the median). (D) The distribution of cell-specific p-values for two-sided and one-sided Wilcoxon hypothesis tests of openness scores of silencers between HepG2 cell line and other 198 cell types (significantly lower than 0.05).

Fig. S3. OpenannotateApi is integrated into the analysis pipeline of Signac.

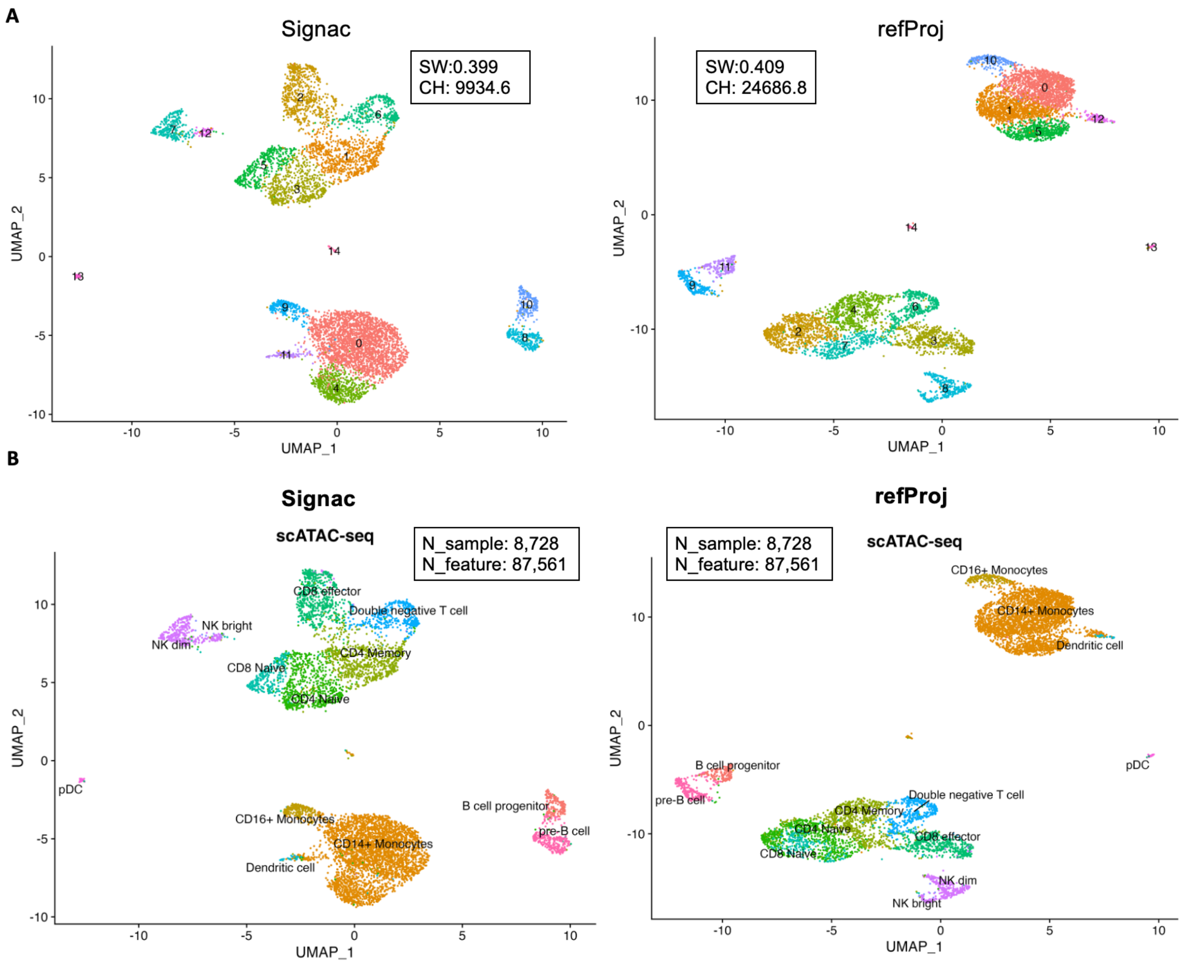

Fig. S3. OpenannotateApi is integrated into the analysis pipeline of Signac. (A) Dimensionality reduction and visualization results on a scATAC-seq dataset containing 8,728 human peripheral blood mononuclear cells with 87,561 features. The left side displays the outcomes from Signac, while the right side presents the clustering results obtained by combining the dimensionality reduction of refProj with that of Signac. The evaluation metric for clustering is the average silhouette width (SW) and Calinski-Harabasz index (CH). (B) Cell type annotation through clustering based on the integration with paired scRNA-seq data.

Fig. S4. OpenannotateApi is integrated into the analysis pipeline of ArchR.

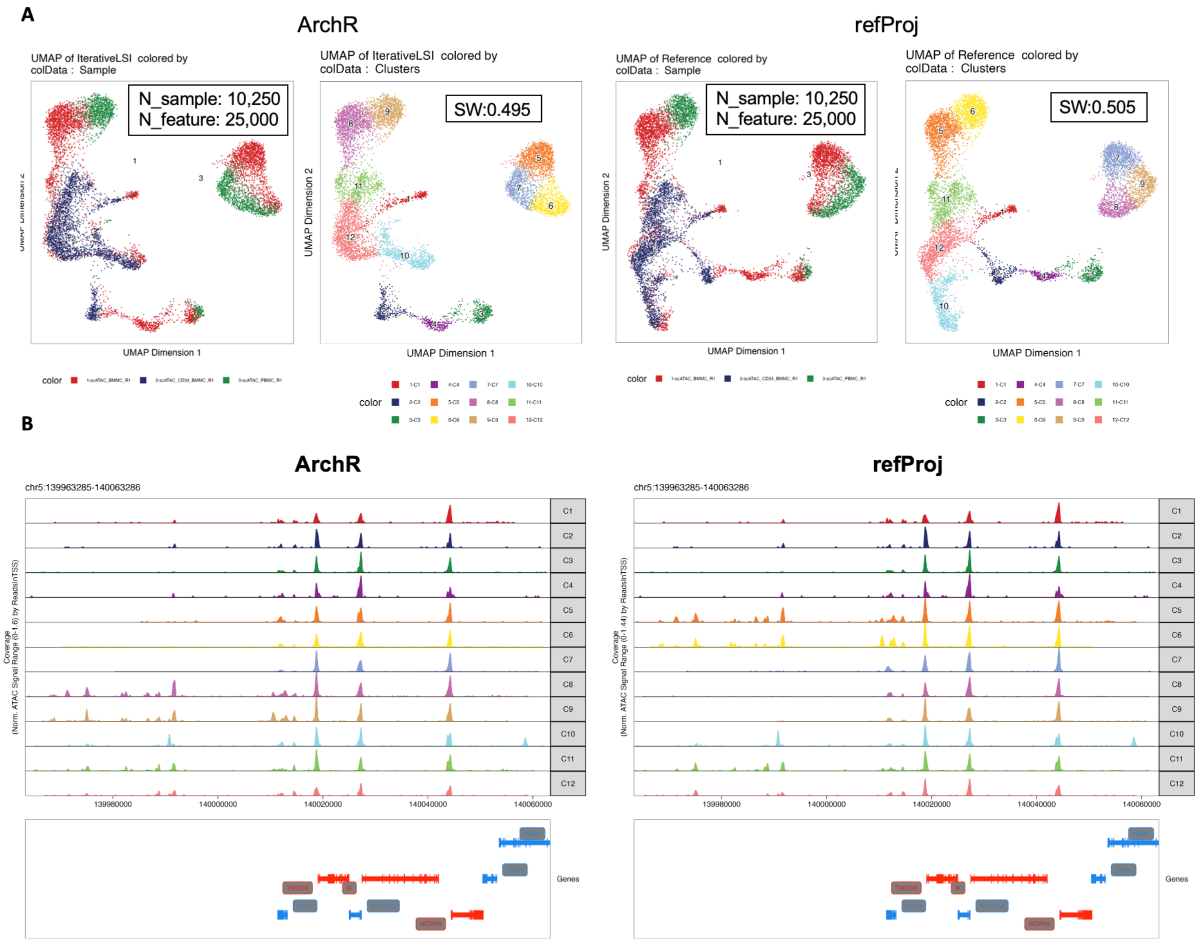

Fig. S4. OpenannotateApi is integrated into the analysis pipeline of ArchR. (A) Dimensionality reduction and visualization results on tutorial data (10,250 cells and 25,000 features). The left side visualized the cell embeddings from ArchR, while the right side denotes the clustering results obtained by combining the cell embedding of refProj with that of ArchR. The evaluation metric for clustering is the average silhouette width (SW). (B) Chromatin accessibility at CD14 marker gene on a per cluster basis with genome browser tracks.

Fig. S5. Computational efficiency of OpenAnnotateApi.

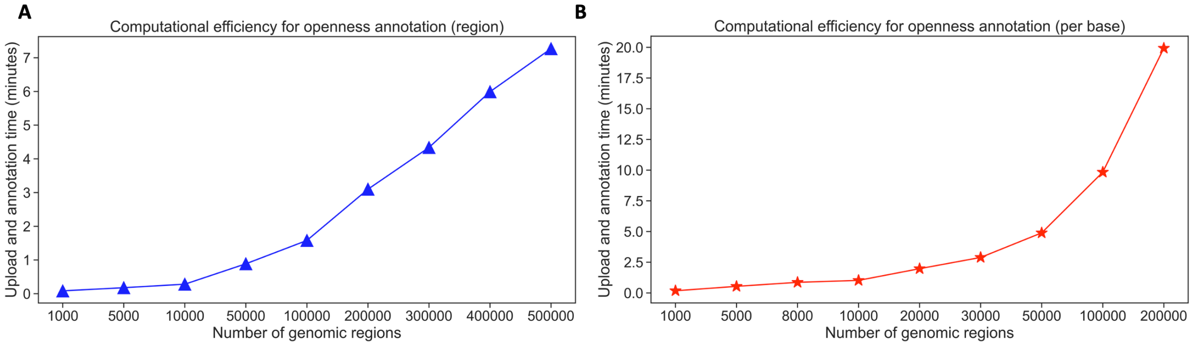

Fig. S5. Computational Efficiency of OpenAnnotateApi. (A) Region-based annotation computational efficiency. We utilized the candidate silencer elements in SilencerDB, where the x-axis denotes the logarithmically transformed number of regions ranging from 1000 to 500,000, and the y-axis denotes annotation time (minutes). (B) Base-pair-based annotation computational efficiency, where the x-axis denotes the logarithmically transformed number of regions ranging from 1000 to 200,000, and the y-axis denotes annotation time (minutes).

Fig. S6. The performance of DeepCAPE and DeepCAPE (seq-only) in predicting enhancers in epithelial cells of the esophagus.

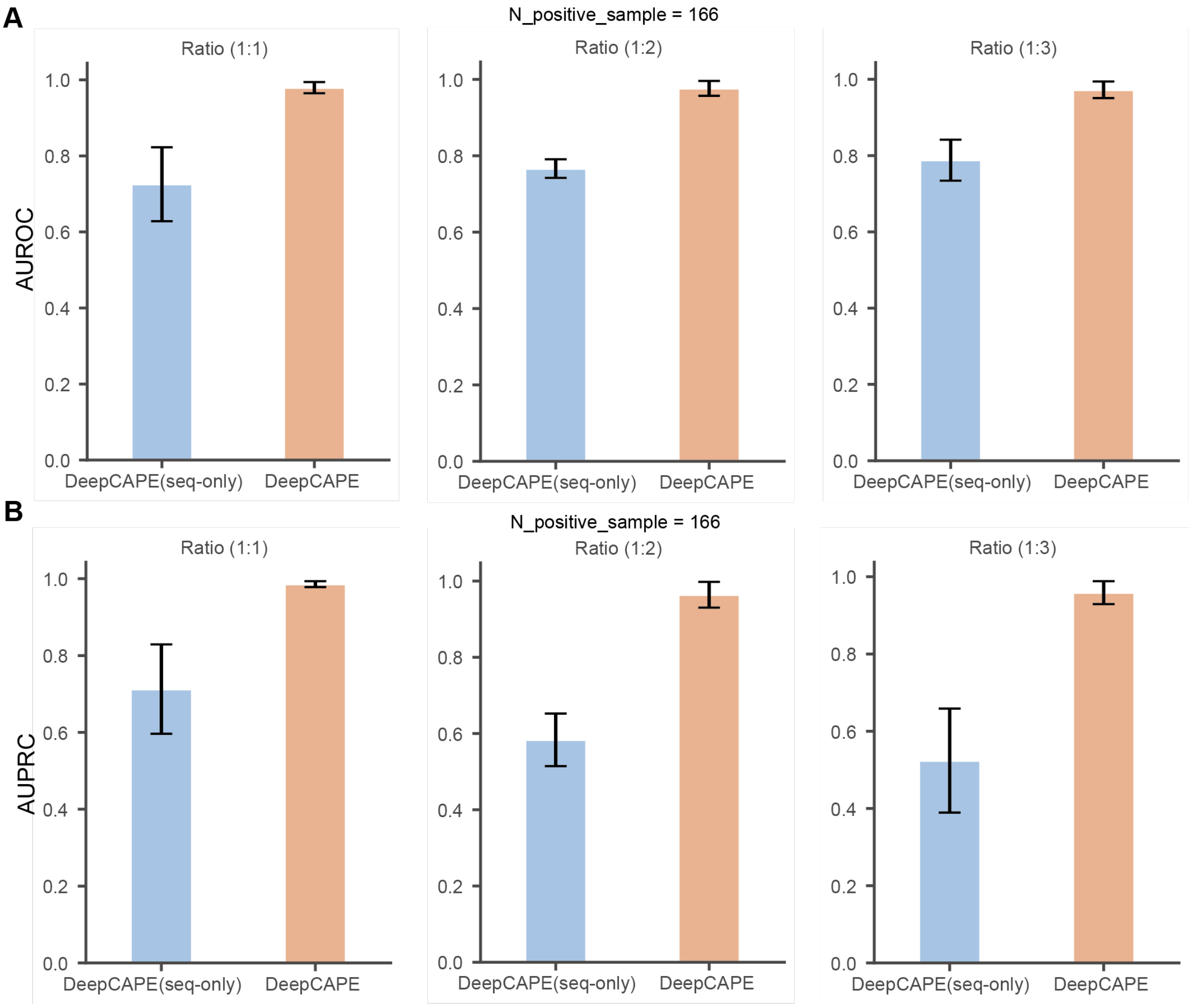

Fig. S6. The performance of DeepCAPE and DeepCAPE (seq-only) in predicting enhancers in epithelial cells of the esophagus. (A). The auROC for predicting enhancers in five-fold cross-validation at sample ratios of 1:1, 1:2, and 1:3, with 166 positive samples. (B). The auPRC for predicting enhancers in five-fold cross-validation at sample ratios of 1:1, 1:2, and 1:3, with 166 positive samples.

Fig. S7. The distribution of the time taken for manually submitting annotation tasks to the web server 20 times (seconds).

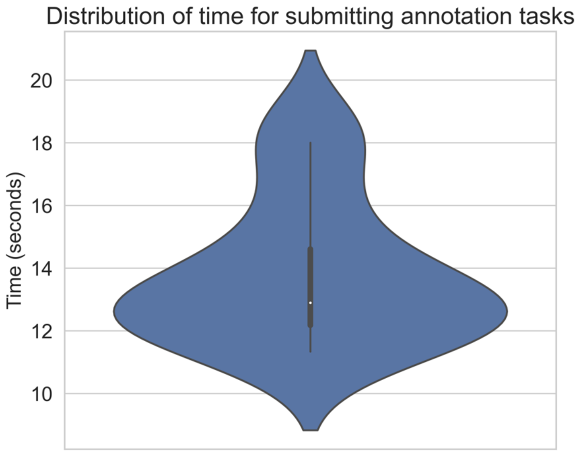

Fig. S7. The distribution of the time taken for manually submitting annotation tasks to the web server 20 times (seconds).

Supplementary Tables

Table S1. Function names and functionalities of OpenAnnotatePy (As of Apr. 2024).

Code	Function
testWebserver()	test whether the web server is working normally
setAddress(IP, port)	set the address of the web server
help()	get a list of the various functions and arguments that the package contains.
getParams()	get params list
getCelltypeList(protocol, species)	get cell types for annotation
getTissueList(protocol, species)	get tissue for annotation
getSystemList(protocol, species)	get systems for annotation
searchCelltype(protocol, species, keyword)	search for cell types that contain keyword
searchTissue(protocol, species, keyword)	search for cell types that contain keyword
searchSystem(protocol, species, keyword)	search for cell types that contain keyword
setParams(assay, species, cell_type, perbase)	set parameters
runAnnotate(input)	upload file to server
getProgress(task_id)	view the annotation progress
getAnnoResult(result_type, task_id, cell_type)	download the annotation result
getInputFile(save_path, task_id)	get input file from server
viewParams(task_id)	view parameters
getExampleTaskID()	get example task id
getExampleInputFile(save_path)	get example input file to the save_path
fromOpen2EpiScanpy(data_path, head_path)	generate anndata from annotation result

Table S2. Function names and functionalities of OpenAnnotateR (As of Apr. 2024).

Code	Function
help()	get a list of the various functions and arguments that the package contains.
getParams()	get params list
getCelltypeList(protocol, species)	get cell type list
getTissueList(protocol, species)	get tissue for annotation
getSystemList(protocol, species)	get systems for annotation
searchCelltype(protocol, species, keyword)	search for cell types that contain keyword
searchTissue(protocol, species, keyword)	search for tissues that contain keyword and corresponding cell types
searchSystem(protocol, species, keyword)	search for systems that contain keyword and corresponding cell types
runAnnotate(file_path, species, protocol, cell_type, perbase)	upload file to server
getProgress(task_id)	view the annotation progress
getAnnoResult(result_type, cell_type, task_id)	download the annotation result
getInputFile(save_path, task_id)	get your input file from server
viewParams(task_id)	view parameters
getExampleTaskID()	get example task id
getExampleInputFile(save_path)	get example input file to the save_path
Openness2Seurat(file_path)	transform annotation results into an R data structure compatible with the Seurat object.

Table S3. Comparison of functionalities in OpenAnnotateApi, OpenAnnotate and other toolkits.

Functionalities, data and applications	OpenAnnotateApi	OpenAnnotate	Cistrome DB/Toolkit	ATACdb	ENCODE /SCREEN	DeepBlue
ATAC-seq	✓	✓		✓	✓	✓
DNase-seq	✓	✓	✓		✓	✓
Annotation for batch genomic regions	✓	✓				
Hierarchical categorization	✓	✓		✓	✓	✓
Query a given genomic region	✓	✓	✓	✓	✓	
Comparison across biosample types		✓	✓		✓	
Annotation in a specific biosample type	✓	✓			✓	
Per-base pair annotation	✓	✓				
Ultra-efficient calculation	✓	✓				
Standardized annotation results	✓	✓				
Real-time browsing of massive results		✓				
Retrieve results by task ID	✓	✓				
Applied to cell type-specific studies	✓	✓				
Incorporated into computational models	✓	✓				
Used as reference for single-cell analyses	✓	✓				
Query a given biological system with keyword	✓					
Query a given tissue with keyword	✓					
Query a given cell type with keyword	✓					

Annotation in multiple biosample types	✓	
Retrieve task parameters by task ID	✓	✓
Convert openness scores to Seurat object	✓	
Convert openness scores to Ann-data format	✓	
Noncoding variants analysis	✓	✓
Gene regulatory mechanism analysis	✓	✓
Integration with single-cell analysis pipeline (Episcanpy)	✓	
Integration with single-cell analysis pipeline (Signac)	✓	
Integration with single-cell analysis pipeline (ArchR)	✓	

Table S4. Summary of the scCAS datasets in single-cell data analyses with OpenAnnotateApi.

Tool	Data name	No. of cells	No. of features	Species
EpiScanpy	MCA_Cerebellum	2,278	436,206	Mus musculus
Signac	PBMC	8,728	87,561	Homo sapiens
ArchR	BMMC/CD43/PBMC	10,660	6,072,620	Homo sapiens

References

Andersson, R., *et al.* (2014) An atlas of active enhancers across human cell types and tissues. *Nature*, **507**, 455-461.

Chen, S., *et al.* (2021) DeepCAPE: a deep convolutional neural network for the accurate prediction of enhancers. *Genomics, Proteomics & Bioinformatics*, **19**, 565-577.

Chen, S., *et al.* (2021) OpenAnnotate: a web server to annotate the chromatin accessibility of genomic regions. *Nucleic Acids Research*, **49**, W483-W490.

Chen, S., *et al.* (2021) RA3 is a reference-guided approach for epigenetic characterization of single cells. *Nature Communications*, **12**, 2177.

Consortium, E.P. (2012) An integrated encyclopedia of DNA elements in the human genome. *Nature*, **489**, 57.

Danese, A., *et al.* (2021) EpiScanpy: integrated single-cell epigenomic analysis. *Nature Communications*, **12**, 5228.

Davis, C.A., *et al.* (2018) The Encyclopedia of DNA elements (ENCODE): data portal update. *Nucleic acids research*, **46**, D794-D801.

Gorkin, D.U., *et al.* (2020) An atlas of dynamic chromatin landscapes in mouse fetal development. *Nature*, **583**, 744-751.

Granja, J.M., *et al.* (2021) ArchR is a scalable software package for integrative single-cell chromatin accessibility analysis. *Nature genetics*, **53**, 403-411.

Granja, J.M., *et al.* (2019) Single-cell multiomic analysis identifies regulatory programs in mixed-phenotype acute leukemia. *Nature biotechnology*, **37**, 1458-1465.

Li, W., *et al.* (2020) A method for scoring the cell type-specific impacts of noncoding variants in personal genomes. *Proceedings of the National Academy of Sciences*, **117**, 21364-21372.

Li, W., *et al.* (2017) Gene co-opening network deciphers gene functional relationships. *Molecular BioSystems*, **13**, 2428-2439.

Li, W., Wong, W.H. and Jiang, R. (2019) DeepTACT: predicting 3D chromatin contacts via bootstrapping deep learning. *Nucleic acids research*, **47**, e60-e60.

Song, S., *et al.* (2019) EpiFIT: functional interpretation of transcription factors based on combination of sequence and epigenetic information. *Quantitative Biology*, **7**, 233-243.

Stuart, T., *et al.* (2021) Single-cell chromatin state analysis with Signac. *Nature methods*, **18**, 1333-1341.

Wang, F., *et al.* (2021) ATACdb: a comprehensive human chromatin accessibility database. *Nucleic Acids Research*, **49**, D55-D64.

Zeng, W., *et al.* (2021) SilencerDB: a comprehensive database of silencers. *Nucleic acids research*, **49**, D221-D228.

Zhang, Z., Chen, S. and Lin, Z. (2023) RefTM: reference-guided topic modeling of single-cell chromatin accessibility data. *Briefings in Bioinformatics*, **24**, bbac540.

Zheng, R., *et al.* (2019) Cistrome Data Browser: expanded datasets and new tools for gene regulatory analysis. *Nucleic acids research*, **47**, D729-D735.
